# Neural mechanisms of affective matching across faces and scenes

**DOI:** 10.1038/s41598-018-37163-9

**Published:** 2019-02-06

**Authors:** Katrin Preckel, Fynn-Mathis Trautwein, Frieder M. Paulus, Peter Kirsch, Sören Krach, Tania Singer, Philipp Kanske

**Affiliations:** 1 Department of Social Neuroscience, Max Planck Institute for Human Cognitive and Brain Sciences, Stephanstraße 1A, 04107 Leipzig, Germany; 2 Edmond J. Safra Brain Research Center for the Study of Learning Disabilities, University of Haifa, Haifa, Israel; 3 Department of Psychiatry and Psychotherapy, Social Neuroscience Lab, Lübeck University, Center of Brain, Behavior and Metabolism (CBBM), 23538 Lübeck, Germany; 4 Department of Clinical Psychology, Central Institute of Mental Health, Medical Faculty Mannheim/Heidelberg University, Mannheim, Germany; 5 Clinical Psychology and Behavioral Neuroscience, Faculty of Psychology, Technische Universität Dresden, Dresden, Germany

## Abstract

The emotional matching paradigm, introduced by Hariri and colleagues in 2000, is a widely used neuroimaging experiment that reliably activates the amygdala. In the classic version of the experiment faces with negative emotional expression and scenes depicting distressing events are compared with geometric shapes instead of neutral stimuli of the same category (i.e. faces or scenes). This makes it difficult to clearly attribute amygdala activation to the emotional valence and not to the social content. To improve this paradigm, we conducted a functional magnetic resonance imaging study in which emotionally neutral and, additionally, positive stimuli within each stimulus category (i.e. faces, social and non-social scenes) were included. These categories enabled us to differentiate the exact nature of observed effects in the amygdala. First, the main findings of the original paradigm were replicated. Second, we observed amygdala activation when comparing negative to neutral stimuli of the same category. However, for negative faces, the amygdala response habituated rapidly. Third, positive stimuli were associated with widespread activation including the insula and the caudate. This validated adaption study enables more precise statements on the neural activation underlying emotional processing. These advances may benefit future studies on identifying selective impairments in emotional and social stimulus processing.

## Introduction

Amygdala functioning is of high interest for clinical psychology, psychiatry and neuroscience, as heightened amygdala activation has been reported in various patient groups^1–5^. The emotional matching paradigm by Hariri *et al*.^6^ and its extended version^7^ are widely used as emotional reactivity measures, which reliably activate the amygdala^8–10^. Despite its current use in psychiatry, this paradigm has a potential drawback since faces with negative emotional expressions and negative social scenes are compared with simple geometric shapes. Thus, it compares pictures that differ in more than one domain: social content and emotional valence. It is therefore difficult to draw conclusions about which of the two different domains causes the increase in amygdala activation. This differentiation may arguably not be relevant for all purposes, but to study specific populations, such as patients with deficits in one or the other domain (e.g. those with autism spectrum disorder (ASD))^11,12^, it is crucial to distinguish the two.

A second issue is that negative emotions have been studied more widely than positive emotions, as exemplified by the original emotional matching paradigm, putatively due to their high functional value for action. For example previous research suggests that threatening scenes, whether they contained faces or no human features, elicited activation in the extrastriate body area, suggesting that this activity to threatening scenes, represents the capacity of the brain to associate certain situations with threat, in order to prepare for fast reactions^13^. Positive emotions are, however, the other side of the coin, as they allow psychological growth and well-being^14^. Positive stimuli are most commonly used in the context of reward experiments, for example in performance-based feedback tasks^15,16^. A brain region that has been related to the processing of various reward types, ranging from primary reinforcers to more abstract social rewards^17,18^, is the ventral striatum^19^. Also, meta-analytically, the ventral striatum elicits the strongest activation across the different reward types such as monetary, food and erotic rewards^20^. The amygdala was also found to be activated in response to positive stimuli. For direct comparisons of positive and negative faces, not all studies found amygdala activation differences^21^, but a meta-analysis of positive and negative affect revealed that the amygdala is more strongly activated for negative stimuli^22^. Other brain regions involved in positive emotion processing include the ventrolateral prefrontal cortex,^23^, medial prefrontal cortex, the insula, the superior temporal sulcus and the inferior frontal gyrus^24^, the cuneus, the inferior occipital lobule, the inferior parietal gyrus, the caudate and the temporal gyrus^20^.

Although probing mean brain activation is the most straightforward attempt to analyze brain data, another important characteristic of the amygdala is its quick habituation^25^. Habituation effects in the amygdala have been found to have a stronger test-retest reliability than mean activation analysis. Plichta and colleagues^26^ found strong retest reliability in the bilateral amygdala for negative faces across two sessions on the group but not on the within-subject level, and they demonstrate in a follow-up article, that amygdala habituation findings pose a better within-subject measure than mean activation amplitudes, because amygdala habituation findings are associated with the highest retest reliability^27^. To potentially treat amygdala activation as a biomarker for psychiatric disorders, it is essential that its activation measures are stable over time in the sense of reflecting a characteristic of the individual. Furthermore, differences in habituation may be more informative than mean activation itself^28^, because habituation is an important adaptive process which reduces responding to irrelevant stimuli^29^. In healthy populations, amygdala habituation to faces occurs very quickly, while in patients with posttraumatic stress disorder, who display an increased amygdala response to fearful faces in comparison to healthy controls (HCs), amygdala habituation to fearful faces is reduced^30^. In ASD patients, amygdala habituation to emotional faces is absent^31^. Furthermore, increased (physiological) habituation is more strongly associated with viewing negative stimuli as compared to viewing neutral stimuli in the healthy population^32^.

To gain a broader understanding of *what* causes amygdala activation (i.e., emotional valence, salience, social or non-social stimulus content)^7^ we expanded the original task version and included more categories and carefully chosen control stimuli. Specifically, we presented three different stimulus categories, distinguishing faces, from social and non-social scenes. And we presented negative, positive and neutral stimuli within each of these three categories. All neutral stimuli act as appropriate controls for the respective condition. Comparable to the low-level control condition in the original matching task, the neutral non-social scenes serve additionally as control stimuli for all emotional conditions in the analyses that aim to replicate the original findings. This full set of stimuli allows us to evaluate whether typically observed amygdala activation are due to faces, the social versus non-social value of the stimuli, or their respective emotional valence. Participants’ task was to decide which of the two pictures presented on the bottom, was identical to a third picture that was simultaneously presented on the top of the screen.

We analyzed behavioral as well as functional magnetic resonance imaging data, with a particular focus on the amygdala. The region of interest (ROI) approach is based on the original Hariri paradigm, which focused exclusively on the amygdala, and the strong meta-analytic data showing amygdala involvement in negative emotion processing. In a first step, we aimed at replicating the findings of the original emotional matching task by computing contrasts that are comparable to the ones reported by Hariri *et al*.^7^. These comparisons contrast negative faces and negative social scenes to neutral non-social pictures and we expected to find amygdala activation in both contrasts. Second, we expected elevated amygdala activation for negative faces and negative scenes compared to their respective within-stimulus category control conditions: neutral faces and neutral social and non-social scenes. This would indicate that negative emotion, not social content, is the driving force of the observed amygdala activity. Regarding the more recent discussions on habituation effects, we hypothesize that amygdala habituation is strongest for negative face pictures as it shows stronger sensitivity to negative affect^22^ and habituates specifically to faces^27^. Third, concerning positive stimuli, we predict stronger neural activation in the ventral striatum, when compared to neutral or negative stimuli. Amygdala activity may also be increased for positive stimuli, but to a smaller degree than for negative stimuli.

## Results

### Behavioral data

All hit rates, describing the rate of correctly matched pictures, were above chance level (Ps < 0.001), it can therefore be assumed that the task was carried out correctly. The mean reaction times (RTs) and hit rates for each category are presented in Table 1. The Shapiro-Wilk test revealed that all RTs are normally distributed (all Ps > 0.05).

**Table 1:** Reaction times and hit accuracy per condition.

Condition	Negative	Neutral	Positive
*Faces*			
Correct hits in %	92	95	95
Reaction times	1032.4 (155.3)	1011.4 (165.9)	1026.1 (159.9)
*Social Scenes*			
Correct hits in %	99	89	98
Reaction times	893.4 (136.1)	855.4 (139.3)	878.4 (140.3)
*Non-social Scenes*			
Correct hits in %	96	83	98
Response times	849.3 (127.8)	774.5 (137.9)	898.6 (134.5)

Table 1 presents hits in % and mean reaction times and standard deviations (SDs) in correct trials in milliseconds.

Even though hit rates were generally very high, a Friedman test revealed a statistically significant difference in accuracy depending on sociality and emotion, χ^2(2)^ = 106.764, P < 0.001, as well as on specific category, χ^2(2)^ = 163.644, P < 0.001. Post hoc analyses with Wilcoxon signed-rank tests resulted in significant differences between the social and non-social conditions (Z = −4.457, P < 0.001), between the non-social and face conditions (Z = −3.431, P = 0.001) and between the social scenes and face conditions (Z = −3.535, P < 0.001). Further, significant differences between negative and positive conditions (Z = −3.024, P = 0.002), between neutral and negative conditions (Z = −4.544, P < 0.001) and between neutral and positive conditions (Z = −4.527, P < 0.001) were observed.

For reaction times, an ANOVA was calculated. Two main effects for RTs were found. How quickly the stimulus pictures were matched correctly, depended on social content (F_(2,52)_ = 82.92; P < 0.001, ηp^2^ = 0.761), as well as on emotional valence (F_(2,52)_ = 33.45, P < 0.001, ηp^2^ = 0.563). Non-social scenes were matched significantly faster than social scenes and social scenes were matched faster than faces (Ps < 0.05). Neutral pictures were matched faster than negative and positive pictures. A significant interaction was found for social content × emotional valence (F_(4,104)_ = 15.12, P < 0.001, ηp^2^ = 0.368). For social scenes, neutral stimuli were matched faster than negative and positive stimuli, while for non-social scenes, neutral pictures were matched faster than negative pictures, but negative pictures were also matched faster than positive non-social scenes.

### fMRI Results

#### Validation of paradigm

To validate the paradigm, the comparable contrasts to those from the original emotional matching paradigm were calculated, that is, negative faces and negative social scenes versus non-social neutral stimuli (cf. Hariri *et al*. 2002). As presented in Table 2, these contrasts yielded the expected significant activation in the bilateral amygdala within the anatomically pre-defined region of interests (ROIs).

**Table 2:** Validation contrasts to the original emotional matching paradigm.

Regions	Coordinates xyz	Cluster size	T-value	(Z_E_)	P-value
**negative faces > neutral non-social scenes**					
Right amygdala (ROI)	21 −3 −12	3	4.50	3.83	0.003
Left amygdala (ROI)	−18 −3 −12	1	3.76	3.33	0.018
**negative social scenes > neutral non-social scenes**					
Right amygdala (ROI)	24 −6 −15	28	6.78	5.10	<0.001
Left amygdala (ROI)	−21 −6 −12	19	5.87	4.64	<0.001

The two contrasts presented in Table 2 are comparable to those from the original emotional matching paradigm, that were negative faces/negative scenes > control. ROIs were defined with the WFU Pickatlas. Small volume corrections were performed. All contrasts were thresholded at a P < 0.05 FWE-corrected level.

#### Amygdala responses to negative emotions versus adjusted control conditions

In this section, we report the comparisons within social categories. Significant bilateral amygdala activation was found for negative social and non-social scenes compared to neutral social and neutral non-social scenes, respectively. For negative faces versus neutral faces, however, no significant difference was observed. Additional whole-brain analyses showed several further activation clusters, including activity in parts of the occipital cortex. The amygdala activation findings, as well as the whole brain findings, of negative faces versus neutral faces and negative social and non-social scenes versus their respective neutral control condition, are presented in Table 3.

**Table 3:** Basic comparisons of negative > neutral stimuli within the same social category.

Regions	Coordinates xyz	Cluster size	T-value	(Z_E_)	P-value
**negative faces > neutral faces**					
Inferior Occipital Gyrus	−30 −87 −6	553	8.31	5.75	<0.001
Fusiform Gyrus	27 −81 −9	125	7.75	5.53	0.001
**negative social scenes > neutral social scenes**					
Amygdala (ROI)	33 0 −30	6	4.43	3.79	0.005
Amygdala (ROI)	−18 −3 −15	4	3.73	3.31	0.023
Amygdala (ROI)	24 −3 −21	4	3.66	3.26	0.026
Middle Occipital Gyrus	−48 −72 6	182	11.63	6.83	<0.001
Superior Temporal Gyrus	51 15 −30	105	9.16	6.07	<0.001
Inferior Frontal Gyrus	39 21 21	54	8.26	5.74	<0.001
Middle Temporal Gyrus	54 −63 6	76	7.87	5.58	0.001
Posterior Cingulum	3 −57 30	36	7.37	5.37	0.002
Inferior Frontal Gyrus	54 33 0	19	7.32	5.34	0.002
Anterior Cingulate	6 −30 −6	4	7.11	5.25	0.004
Superior Temporal Gyrus	−45 18 −27	1	6.23	4.83	0.024
**negative non-social scenes > neutral non-social scenes**					
Amygdala (ROI)	21 −3 −15	5	3.95	3.46	0.014
Amygdala (ROI)	−21 −6 −12	2	3.59	3.21	0.030
Parahippocampal Gyrus	27 −45 −6	2150	16.29	Inf	<0.001
Precuneus	18 −51 12	80	8.51	5.83	<0.001
Calcarine	−18 −57 12	13	7.41	5.39	0.002
Thalamus	−21 −27 −3	5	6.54	4.98	0.012

In Table 3 amygdala activation in the pre-defined ROIs, as well as the whole brain findings for the comparisons of negative > neutral stimuli within the same social category are displayed. ROIs were defined with the WFU Pickatlas. All contrasts were thresholded at a P < 0.05 FWE-corrected level. Abbreviations: region of interest (ROI), non-significant (n.s.).

#### Brain activation to positive emotions versus the adjusted control conditions

No increased brain activation for positive emotions as opposed to neutral or negative emotions was found in the ventral striatum. However, the caudate nucleus, as part of the dorsal striatum, showed stronger activation for positive versus negative pictures on a whole brain level. Details on neural activation for these findings are presented in Table 4.

**Table 4:** Mean brain activation for positive emotion.

Regions	Coordinates xyz	Cluster size	T-value	(Z_E_)	P-value
**positive > neutral**					
Cuneus	15 −99 6	568	12.05	6.94	<0.001
Cuneus	−18 −96 −3		11.46	6.78	
Middle Occipital Gyrus	−21 −99 9		11.12	6.69	
Inferior Occipital Gyrus	39 −63 −12	15	7.49	5.42	0.001
Brain stem	6 −33 −12	2	6.79	5.10	0.008
Brain stem	−6 −27 −15	1	6.44	4.94	0.017
Vermis	−3 −36 −12	2	6.16	4.79	0.031
**positive > negative**					
Insula	33 12 15	6	7.16	5.28	0.003
Inferior Parietal Lobule	54 −48 39	10	6.28	4.86	0.022
Caudate	−21 15 12	3	6.18	4.81	0.027
Medial Frontal Gyrus	6 33 33	1	5.98	4.70	0.042
Temporal Lobe	−33 −42 12	1	5.97	4.69	0.043

In Table 4 the brain areas that elicited stronger activation for positive > neutral and positive > negative stimuli on a whole brain level are presented. All contrasts were thresholded at a P < 0.05 FWE-corrected level.

Amygdala activation was also explored for the contrasts of positive versus neutral conditions for all social content categories, but did not reach significance in any social domain. Comparisons of negative social and non-social scenes versus positive social and non-social scenes did show stronger amygdala activation for the negative conditions. Results can be found in Table S1 (Supplementary Material).

#### Amygdala habituation effects

**Figure 1:**
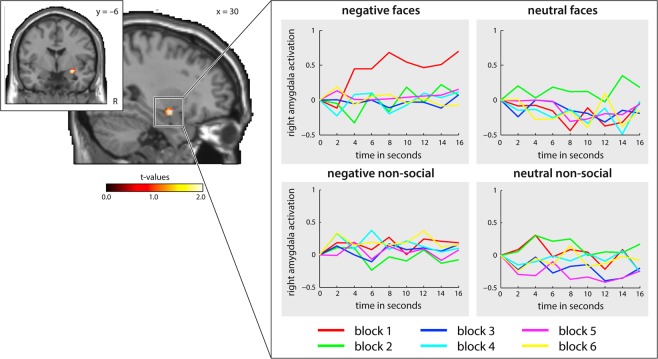
The time course of amygdala activation for negative (upper left) and neutral (upper right) faces, as well as for negative non-social (lower left) and neutral non-social scenes (lower right) across the different blocks is presented. Block 1 is shown in red, block 2 in green, block 3 in dark blue, block 4 in light blue, block 5 in pink and block 6 in yellow.

Significant habituation effects for negative and neutral faces were found in the amygdala in a first minus last block analysis (see Table 5) as previously applied by Plichta *et al*.^27^. As can be seen in the activation time course in the amygdala for negative and neutral faces (see Fig. 1), amygdala activation is highest in the first block of negative faces, which is significant when comparing activation within the first blocks only (see Table 5, there was no significant effect when comparing the last blocks only). Furthermore, an interaction between the first and the last block and the emotional category was found: amygdala habituation to negative faces was significantly stronger than amygdala habituation to neutral faces.

**Table 5:** First > last block amygdala habituation findings for fearful and neutral faces.

Regions	Coordinates xyz	Cluster size	T-value	(Z_E_)	P-value
**negative faces first block > neutral faces first block**					
Amygdala (ROI)	30 −6 −12	14	4.57	3.88	<0.001
**negative faces first > last block**					
Amygdala (ROI)	24 −6 −12	15	5.79	4.60	<0.001
Amygdala (ROI)	−21 −6 −12	3	3.73	3.31	0.019
**neutral faces first > last block**					
Amygdala (ROI)	24 −6 −15	13	4.46	3.93	0.003
Amygdala (ROI)	−24 −9 −15	4	4.03	3.52	0.010
**(negative faces first > last block) > (neutral faces first > last block)**					
Amygdala (ROI)	30 −6 −12	4	3.97	3.48	0.012

In Table 5 the amygdala ROI findings for the first minus last block habituation effects of negative and neutral faces are displayed. ROIs are designed with the WFU Pickatlas. All contrasts were thresholded at a P < 0.05 FWE-corrected level.

No amygdala habituation effects were found for any of the other categories. The respective time course series are presented in the supplementary material (Supplementary Figure S3). Whole brain habituation findings for the first minus last block analysis are presented in the Supplementary Table S4.

In our study, neither linear parametric modulation (Pmod), nor logarithmic parametric modulation^27^ could explain the relationship of amygdala habituation between blocks for negative faces (for details about the other conditions, please see the section about *Habituation Analysis* in supplementary material and Tables S2 and S3).

## Discussion

The present study successfully validated and extended the well-known and widely used emotional matching fMRI paradigm. The new neutral control stimuli, the additional positive emotional condition and the habituation analysis of the amygdala response to negative faces provide further insights into the relationship of (social) emotion processing and amygdala functioning.

The validation contrasts that are comparable to those in the original publication (negative faces and scenes >non-social neutral stimuli) resulted in bilateral amygdala activation. Comparisons within the social categories also yielded significant bilateral amygdala activation for negative social and non-social scenes compared to neutral social and non-social scenes, respectively. We observed no overall amygdala activation for negative versus neutral faces, but a habituation analysis revealed that the amygdala is activated during the first block and then habituates. Concerning the positive stimuli, we did not find activation in the ventral striatum, but increased activation was observed in various other areas including inferior occipital and parietal regions, the insula, the caudate and the temporal lobe.

The additional stimulus categories of neutral and positive emotional pictures, as well as social and non-social scenes allow further insights on the relationship between different stimulus categories and can explain more precisely what stimulus characteristics contribute to increased amygdala activation.

The data convincingly show that negative social and non-social scenes elicit stronger amygdala activation than neutral control scenes of the same category confirming previous claims of negative valence eliciting amygdala activation. Therefore, negative valence seems to have a strong influence on amygdala activation, even though additional explanatory influences such as salience or arousal cannot be ruled out. Against expectations, for the face conditions, the comparison of negative versus neutral did not result in significant overall amygdala activation. This was surprising, given the vast amount of literature showing that the amygdala responds particularly strongly to negatively valenced facial expressions^33–37^. However, also other studies that have conducted the comparison of fearful versus neutral faces^38^ and fearful body postures versus neutral body postures did not find amygdala activation^39^. The results of the habituation analysis do, however, offer a reasonable explanation for the missing overall amygdala activation for negative emotional faces. The analysis of the first versus last block revealed that the amygdala habituates significantly more to negative than to neutral faces; the negative versus neutral contrast was only significant in the first, not the last blocks. In contrast to the first versus last block habituation findings, parametric modulation effects did not explain amygdala habituation to negative faces; neither the linear nor the logarithmic habituation approaches can explain the relationship of habituation to negative faces in the amygdala. This suggests very rapid habituation to negative faces, which reaches a plateau already after the first block and does not decrease further. The first versus last block habituation results are consistent with previous findings that also observed significant amygdala habituation to negative face stimuli^27^. Our results of an interaction in the amygdala for face conditions with activation to negative faces habituating more strongly than to neutral faces are at odds, however, with those of Tam *et al*.^31^. The lack of an interaction in this study may be explained by the applied experimental task. Tam *et al*.^31^, used an n-back task in which three types of emotional faces (neutral, angry, happy) were presented in addition to scrambled images as distractors. The emotional faces were not tested for memory, but a letter that was presented between two identical faces. Our task had qualitatively different negative faces (fearful instead of angry) and these faces, as well as the other stimuli were the direct focus of attention for participants. Moreover, rapid amygdala habituation to negative faces has some plausibility, when considering evolutionary explanations. Stimuli which initially result in aversive responses are no longer perceived as aversive once the participant has learned that these stimuli do not pose any threat. Stimuli that are not threatening at any time do not require re-evaluation and consequently no habituation^29,40^. The specific habituation to fearful faces when compared to threatening scenes might be explained by the higher similarity of the fearful faces amongst each other. Threatening scenes are more diverse and might pose different threats to the observer that would require a different type of habituation for each scenes. This would be an interesting and important question to investigate for future studies.

Positive affective stimuli did not result in amygdala activation when compared to neutral stimuli, which suggests that the amygdala responds strongest to *negative* affective stimuli. The neural activation observed for positive stimuli is not in accordance with our expectations, because we did not find ventral striatal activation. The function of ventral striatal activation is strongly debated, but most frequently linked to reward processes, either in the context of reward prediction (errors) or reward value processes^41,42^. The previously mentioned meta-analysis^20^ revealed that the strongest ventral striatal activation resulted from monetary rewards as compared to erotic and food rewards. The authors suggested that this might be due to the nature of the applied monetary reward paradigms, rather than to monetary rewards themselves, because monetary reward paradigms usually involve stimulus-reward associations. The other positive stimuli that were investigated in that meta-analysis, such as erotic and food pictures, were mostly used in passive viewing paradigms. Additionally, stronger ventral striatal activation was elicited when stimuli were presented unexpectedly^43^ or during the anticipation of a reward^44^. The study at hand is more closely related to the passive viewing paradigms, which contributed less to ventral striatal activation. Moreover, the block design prevents unexpectedness, at least to some extent, because four pictures of the same category are always presented in a row. These experimental characteristics might explain the missing activation in the ventral striatum to the positive versus neutral stimuli in our study. However, even though we did not find ventral striatal activity, we did find other regions such as the cuneus, inferior occipital and parietal regions, the insula, part of the dorsal striatum (caudate) and the temporal lobe, that have also been reported in prior studies investigating reward processing^20^. The large network of brain areas that was activated when positive stimuli were shown is consistent with meta-analytic findings, suggesting that our results are plausible. Even though the insula is predominantly associated with negative stimuli^22^, it also mediates approach and avoidance behavior when social affective stimuli^45^ are presented and the positive stimuli in this study are chosen for their affective and affiliative nature.

There are several other adaptation experiments of the original Hariri paradigm (2002), for example by Paulus and colleagues^46^, who focused on emotional matching, thereby presenting three different facial identities from which two identities expressed the same emotion, and they continued to use geometrical shapes as control stimuli. Other studies used different designs, with presenting dynamic faces^47^, or negative and neutral pictures with human or nonhuman content^48^, investigating brain systems which are involved in social emotion perception and regulation. In this study, we tried to stick very closely to the original Hariri paradigm to ensure comparability between the two experimental designs and simultaneously eliminate important confounds which we think was successful. However, two major limitations of the study should be addressed. Firstly, a block design is not the best approach to measure habituation effects. We chose to use the block design, nonetheless, because our main goal was to validate and extend an existing paradigm, which was originally built as a block design and which has already been successfully studied with respect to habituation effects^27^. Furthermore, the different arousal ratings for the three affective stimuli categories might be a potential confound and in part account for the amygdala activation^49^. However, we chose low arousal positive stimuli on purpose, because we wanted to investigate affectionate neural activations.

This study closes an important gap by providing more appropriate control stimuli for the differentiation of emotional valence versus social content effects on amygdala activation. Our adapted paradigm could be validated by replicating the contrasts of the original emotional matching paradigm, and successfully improved some shortcomings. These advances may benefit future studies aiming to identify selective impairments in social emotional processing in psychiatric conditions.

Furthermore, we would like to note that the sample size was relatively small and included only male participants. While this was a conscious choice since this study served as a pilot study for clinical trials with male participants only^11,12^. Future studies should however investigate this extended paradigm with a larger sample, also including female participants.

## Methods

### Participants

Thirty-two men, from which five participants had to be excluded, because of missing data, participated in this study after giving written informed consent. Thus, data from 27 men aged between 22 and 35 years (M = 28.78, SD = 3.41), could be analyzed. The study was approved by the ethics committee of the University of Leipzig and was conducted in accordance with the Declaration of Helsinki. This study served as a pilot study for clinical trials involving male ASD patients and neurotypical males, therefore, only male participants were included in this pilot study. For a detailed description of the clinical trials, please see^11,12^.

### Acquisition of fMRI Data

Functional magnetic resonance imaging (fMRI) data was acquired for two runs, using a 3 T Siemens Verio scanner (Siemens Medical Systems, Erlangen), equipped with a 32-channel head coil. T2*-weighted echoplanar images with blood-oxygen-level-dependent contrast were obtained (TR = 2 s, TE = 27 ms, matrix size = 70 × 70, number of slices = 37, slice thickness = 3 mm, FoV = 210, flip angle = 90°). A high quality T-1 weighted image was available from the in-house database (TR = 2.3 s, TE = 2.98 ms, slices = 176, slice thickness = 1 mm, voxel volume = 1 × 1 × 1 mm, FOV = 256 mm^2^, flip angle = 9°) for each participant so that no additional T-1 weighted image for anatomical reference was acquired.

### Procedure

After completing a practice trial to ensure that instructions were correctly understood, the participant was placed into the scanner. While lying inside the scanner, a beamer projected stimuli onto a screen and the participant viewed the images through a mirror mounted on the head-coil. Stimuli were presented with the Presentation Software (Neurobehavioral Systems, Albany, CA).

The presented picture stimuli were taken from different databases, the International Affective Picture system (IAPS)^50^, the Emotional Picture System (EmoPics)^51^, the Radboud Faces Database^52^ as well as from the internet. Previous to the scanning sessions, these pictures were divided into 9 categories: negative (fearful) faces, neutral faces, positive (happy) faces, negative social scenes (e.g. a crying baby), neutral social scenes (e.g. a man reading a newspaper), positive social scenes (e.g. a smiling couple), negative non-social scenes (e.g. rubbish dumb), neutral non-social scenes (e.g. mugs on a table) and positive non-social scenes (e.g. delicious food). Stimuli were chosen and placed into categories based on their provided valence ratings (social and non-social scenes) or their percentage of agreement on emotion categorization (faces), respectively. Furthermore, pictures belonged to the social categories when people were shown on the pictures. People depicted in the social scenes never looked directly into the camera.

Two stimuli pictures in one three-picture alignment resembled each other as much as possible in their scenery set-up to increase matching difficulty. Participants watched each three-picture alignment and indicated by button press, which of the two pictures presented at the bottom of the screen was identical to the one presented in the middle on top. Four three-picture alignments (henceforth: stimulus pictures) of the same category (e.g. positive social scenes) were presented per block.

### Experimental Paradigm

During the fMRI session, participants performed an adapted version of the emotional matching task^7^. Most importantly, the adaption entailed different picture choices. In this block design, stimuli differed in social content (faces, social and non-social scenes) as well as in emotional valence (negative, neutral, positive). Blocks were presented in a pseudorandomized fashion. Each participant completed two experimental runs. Each run presented a different stimulus picture set to avoid viewing the same stimulus pictures in both runs. A further difference between the two runs was the presentation duration of each block. One run presented 16-second blocks and the other run presented 12-second blocks. Four blocks per category were presented in each session, thus eight blocks per category in total, if no missings occurred (for details on missings please refer to Table S5a–c in the supplementary methods). Each block consisted of four pictures of the same category, e.g. social positive scenes. The order of stimulus set and duration presentation was balanced. In total 9 categories were shown in both runs of this block design. Due to failure of equipment, some data was lost so that each category block was presented 6–8 times instead of each category block being presented 8 times (for details see Supplementary Table S5a–c). The order in which stimulus blocks were presented in each run was alternated to avoid sequence effects. More details on the procedure can be found in the supplementary material.

### MRI Data Analysis

The MRI data were analyzed using SPM8 software (Welcome Trust Centre for Neuroimaging, London, UK; http://www.fil.ion.ucl.ac.uk/spm) implemented in Matlab 8 (The MathWorks, Natick, MA). All volumes were coregistered to the SPM single-subject canonical EPI image, slice-time corrected and realigned to the mean image volume. A high resolution anatomical image of each subject was first coregistered to the SPM single-subject canonical T1 image and then to the average functional image. The transformation matrix obtained by normalizing the anatomical image was used to normalize functional images to MNI space. The normalized images were spatially smoothed using an 8-mm full width at half maximum Gaussian kernel. A high-pass temporal filter with cutoff of 432 s and 512 s, respective to the short or long session, was applied to remove low-frequency drifts from the data.

### Statistical fMRI analysis

Statistical analysis was carried out using a general linear model. Onsets and durations of the 9 conditions block design were modeled with a boxcar function and convolved with a hemodynamic response function^53^. To reduce potential noise-artifacts we used the rWLS toolbox in which a restricted maximum likelihood algorithm estimates the variance of the noise for each image and weighs images in accordance to their variance^54^. Paired t-tests were calculated between the two different runs, to determine whether the collected data from the two runs can be analyzed together or not. This comparison was made for the two different stimuli sets, as well as for the two different block durations. The main objective of this study was to validate our task by testing whether amygdala activation, as observed by Hariri *et al*.^7^, can be replicated with our adapted control stimuli. Therefore, we first calculated the contrasts of interest on the first level for each participant (here: negative faces – neutral non-social scenes), afterwards the parameter estimates for each contrast were used for a one sample t-test on the second level in order to investigate the amygdala activation for this contrast. The ROI analyses of the amygdala were performed by using atlas-based structurally defined masks which were based on amygdala classification of the automatic anatomic labeling (AAL) and Talairach Deamon (TD) templates^55,56^ in the WFU Pickatlas^57^. The threshold we chose was at P < 0.05 (small volume (family-wise error (FWE)) corrected). The second aim was to investigate whether amygdala activation can be observed for negative faces and negative social scenes versus their respective control condition, thus one-sample t-tests were conducted for the respective contrasts the same ways as previously described. Our third aim was to test whether positive stimuli elicit activation in the ventral striatum. A ROI from the WFU Pickatlas was used for the analysis and t-tests were conducted in the same way as previously described for the amygdala and the respective contrasts. Our fourth goal was to test, which stimuli the amygdala habituates to. In order to investigate this, we used the first minus last block analysis, as well as the habituation approach by means of Pmod. The first minus last block analysis investigates the activation amplitude difference between the first and the last block and if a difference is found, it can be claimed that significant habituation has taken place from the first to the last block. The Pmod approach examines the linear relationship of block number and amygdala activation across the whole experiment and if a significant result is obtained, it can be concluded that the habituation of brain activation can be explained by a systematic reduction over time and that the brain amplitude can be predicted by knowing the block number. Our approaches are based on the description by Plichta^27^ and Tam^31^.

The whole brain analysis used one sample t-tests of direct comparisons between two different categories of interest, for example: positive >neutral and positive >negative stimuli on a P < 0.05 FWE-corrected level.

## Supplementary information

Supplementary Information

## Data Availability

The described dataset of the current study is available from the corresponding author on reasonable request.
