# Supplementary material for: Neural mechanisms of affective matching across faces and scenes
Source: Sci Rep. 2019 Feb 6;9:1492. doi: 10.1038/s41598-018-37163-9 (PMC6365558; doi:10.1038/s41598-018-37163-9)

**Supplementary Information for “Neural mechanisms of affective matching across faces and scenes”**

**Authors:**

*Katrin Preckel¹ (preckel@cbs.mpg.de)

Fynn-Mathis Trautwein¹^,^² (mathis.trautwein@gmail.com)

Frieder M. Paulus³ (paulus@snl.uni-luebeck.de)

Peter Kirsch^4^ (peter.kirsch@zi-mannheim.de)

Sören Krach³ (krach@snl.uni-luebeck.de)

Tania Singer¹ (singer@cbs.mpg.de)

Philipp Kanske^1,5^ (kanske@cbs.mpg.de)

*corresponding author

1 Department of Social Neuroscience, Max Planck Institute for Human Cognitive and Brain Sciences Stephanstraße 1A 04107 Leipzig Germany

2 Edmond J. Safra Brain Research Center for the Study of Learning Disabilities, University of Haifa, Israel

3 Department of Psychiatry and Psychotherapy, Social Neuroscience Lab, Lübeck University, Center of Brain, Behavior and Metabolism (CBBM), 23538 Lübeck, Germany

4 Department of Clinical Psychology, Central Institute of Mental Health, Medical Faculty Mannheim/ Heidelberg University, Mannheim, Germany

5 Clinical Psychology and Behavioral Neuroscience, Faculty of Psychology, Technische Universität Dresden Dresden Germany

**Supplementary Results**

***Behavioral results***

*Ratings of stimulus material*

Figure S1A and S1B present the valence ratings, which increase significantly (Ps < 0.01) from negative (social M = 2.84; non-social M = 3.18) to neutral (social M = 5.18; non-social M = 5.16) to positive (social M = 7.06; non-social M = 6.66) in the social stimuli (S1A), as well as in the non-social stimuli (S1B). Negative stimuli, regardless of social context (social or non-social) have been rated as most arousing (social M = 5.8; non-social M = 5.17) followed by positive arousing (social M = 4.53; non-social M = 4.04) and then neutral arousing (social M = 3.12; non-social M = 3.07) pictures (Fig. S1A and S1B).

Because, the pictures taken from the internet did not have ratings, we had the whole picture set rated by male participants (N = 28, mean age = 28.29 years), who did not participate in this study. This was important, because no valence and arousal ratings were present for those pictures that were taken from the internet. As the figures S1 and S2 show, the rating patterns between the “dataset” values and those from our “own ratings” did not change, indicating that additionally chosen pictures fit well into their determined category.

***fMRI Results***

On a neural level, no significant differences were found between the long and the short presentation versions and neither between the two different stimuli sets that were presented.

*Brain activations for negative versus positive stimuli*

Whole brain findings for negative versus positive stimuli are presented in Table S1. The findings are presented separately for each social category.

Table S1: Significant amygdala activation for negative versus positive and positive versus neutral stimuli within the same social category

| **Regions** | **Coordinates xyz** | | **Cluster size** | **T-value** | **(Z_E_)** | **P-value** |
| --- | --- | --- | --- | --- | --- | --- |
| **negative faces > positive faces** | | | | | |  |
| Lingual Gyrus | -9 -84 -6 | | 37 | 6.63 | 5.03 | 0.008 |
| Calcarine | 0 -87 0 | |  | 6.52 | 4.97 | 0.010 |
| Fusiform Gyrus | 24 -78 -12 | | 1 | 5.80 | 4.60 | 0.044 |
| **negative social scenes > positive social scenes** | | | | | | |
| Precuneus | | 15 -54 21 | 32 | 7.55 | 5.45 | 0.001 |
| Superior Temporal Gyrus | | 48 18 -27 | 53 | 7.37 | 5.37 | 0.002 |
| Middle Temporal Gyrus | | 51 6 -21 |  | 7.06 | 5.23 | 0.004 |
| Middle Temporal Gyrus | | 51 -3 -18 |  | 6.91 | 5.16 | 0.005 |
| Posterior Cingulate | | 9 -51 6 | 7 | 6.5 | 4.96 | 0.012 |
| Fusiform | | 27 -33 -18 | 5 | 6.06 | 4.74 | 0.029 |
| Parahippocampal Gyrus | | -24 -42 -9 | 1 | 6.00 | 4.71 | 0.034 |
| Amygdala (ROI) | | 33 3 -21 | 21 | 4.51 | 3.84 | 0.004 |
|  | | 24 -3 -21 |  | 4.34 | 3.73 | 0.005 |
|  | | 33 0 -30 |  | 4.07 | 3.54 | 0.010 |
| Amygdala (ROI) | | -24 -3 -18 | 4 | 3.64 | 3.25 | 0.025 |
| **negative non-social scenes > positive non-social scenes** | | | | | |  |
| Parahippocampa Gyrus | | 27 -45 -6 | 92 | 10.17 | 6.41 | < 0.001 |
|  | | 30 -30 -18 |  | 7.29 | 5.33 | 0.002 |
| Parahippocampa Gyrus | | -30 -39 -12 | 106 | 9.30 | 6.12 | < 0.001 |
|  | | -24 -48 -9 |  | 9.20 | 6.08 | < 0.001 |
| Precuneus | | 24 -57 18 | 52 | 8.46 | 5.81 | < 0.001 |
|  | | 15 -51 12 |  | 6.96 | 5.18 | 0.004 |
| Middle Temporal Gyrus | | -42 -78 18 | 48 | 8.45 | 5.81 | < 0.001 |
| Middle Temporal Gyrus | | 48 -72 18 | 76 | 8.00 | 5.63 | < 0.001 |
|  | | 36 -78 15 |  | 7.57 | 5.45 | 0.001 |
| Amygdala | | -27 -6 -15 | 3 | 6.30 | 4.87 | 0.017 |
| Precuneus | | -6 -54 12 | 14 | 6.29 | 4.86 | 0.018 |
|  | | -18 -57 15 |  | 6.25 | 4.84 | 0.019 |
| Amygdala (ROI) | | -27 -6 -15 | 27 | 6.30 | 4.87 | < 0.001 |
| Amygdala (ROI) | | 21 -3 -15 | 20 | 5.24 | 4.29 | 0.001 |
| Amygdala (ROI) | | 30 3 -27 | 1 | 3.82 | 3.37 | 0.017 |

Table S1 displays the whole brain activation patterns for the comparisons of negative versus positive stimuli within the same social content category. In addition activation for anatomically predefined amygdala ROIs are presented. ROIs were defined with the WFU Pickatlas. All contrasts were thresholded at a P < 0.05 FWE-corrected level.

*Habituation Analysis*

B

Linear parametric modulation did reach weak, but significant activation in the left amygdala for social neutral and positive as well as for the non-social positive scenes (for details see Table S2). However, no amygdala activation could be observed for the remaining six categories. Neither did we find significant amygdala activation for the following contrasts after linear parametric modulation for each condition: negative faces > non-social neutral, social negative > non-social neutral scenes, negative faces > neutral faces, non-social negative > non-social neutral or social negative > social neutral.

Table S2: Significant amygdala habituation after linear parametric modulation

| **Regions** | **Coordinates xyz** | **Cluster size** | **T-value** | **(Z_E_)** | **P-value** |
| --- | --- | --- | --- | --- | --- |
| **non-social positive (linear parametric modulation)** | | | | |  |
| Amygdala (ROI) | -21 -6 -18 | 2 | 3.50 | 3.14 | 0.032 |
| **social neutral (linear parametric modulation)** | | | | |  |
| Amygdala (ROI) | -21 -6 -18 | 1 | 3.49 | 3.13 | 0.032 |
| **social positive (linear parametric modulation)** | | | | |  |
| Amygdala (ROI) | -24 -9 -15 | 1 | 3.35 | 3.02 | 0.041 |

Table S2 presents all significant amygdala findings after conducting linear parametric modulation. ROIs were defined with the WFU Pickatlas. All contrasts were thresholded at a P < 0.05 FWE-corrected level.

Logarithmic “plus 1” (LOG1) resulted in significant left amygdala activation for the following conditions: neutral and positive faces, social negative, non-social neutral and non-social negative scenes. Bilateral significant amygdala activation was found for social neutral, positive and non-social positive scenes. Negative faces was the only condition for which no significant amygdala LOG1 habituation was found (for details see Table S3). The following contrasts did not result in amygdala activation: negative faces > non-social neutral, negative faces > neutral faces, positive faces > neutral faces, negative faces > positive faces, non-social negative > non-social neutral, negative social > neutral social, social negative > non-social neutral and non-social negative > non-social neutral.

Table S3: Significant amygdala habituation after LOG1 parametric modulation

| **Regions** | **Coordinates xyz** | **Cluster size** | **T-value** | **(Z_E_)** | **P-value** |
| --- | --- | --- | --- | --- | --- |
| **neutral faces (logarithmic modulation)** | | | | |  |
| Amygdala (ROI) | -21 -6 -15 | 3 | 3.58 | 3.20 | 0.028 |
| **positive faces (logarithmic modulation)** | | | | |  |
| Amygdala (ROI) | -21 -3 -15 | 1 | 3.44 | 3.09 | 0.034 |
| **social negative (logarithmic modulation)** | | | | |  |
| Amygdala (ROI) | -21 -6 -15 | 3 | 3.54 | 3.17 | 0.028 |
| **non-social neutral (logarithmic modulation)** | | | | |  |
| Amygdala (ROI) | -24 0 -18 | 1 | 3.52 | 3.15 | 0.027 |
| **non-social negative (logarithmic modulation)** | | | | |  |
| Amygdala (ROI) | -21 -6 -18 | 3 | 3.55 | 3.18 | 0.029 |
| **social neutral (logarithmic modulation)** | | | | |  |
| Amygdala (ROI) | 24 -6 -15 | 4 | 3.65 | 3.25 | 0.022 |
| Amygdala (ROI) | -21 -6 -18 | 2 | 3.59 | 3.21 | 0.025 |
| **social positive (logarithmic modulation)** | | | | |  |
| Amygdala (ROI) | -24 -9 -15 | 4 | 3.91 | 3.44 | 0.013 |
| **non-social positive (parametric modulation)** | | | | |  |
| Amygdala (ROI) | -27 0 -15 | 26 | 4.69 | 3.95 | 0.002 |
| Amygdala (ROI) | 30 -6 -15 | 1 | 3.35 | 3.02 | 0.043 |
| Amygdala (ROI) | 21 0 -15 | 1 | 3.29 | 2.98 | 0.049 |

In Table S3 presents all significant amygdala findings after conducting logarithmic parametric modulation. ROIs were defined with the WFU Pickatlas. All contrasts were thresholded at a P < 0.05 FWE-corrected level.

Whole brain results for the FmL habituation analysis are presented in Table S4.

Table S4: Significant whole brain activation after conducting FmL habituation analysis are presented

| **Regions** | **Coordinates xyz** | **Cluster size** | **T-value** | **(Z_E_)** | **P-value** |
| --- | --- | --- | --- | --- | --- |
| **negative faces first > last block** | | | |  |  |
| Middle Occipital Gyrus | 48 -72 0 | 307 | 12.95 | 7.17 | < 0.001 |
| Inferior Parietal Lobule | 30 -48 36 | 90 | 7.89 | 5.59 | 0.001 |
| Superior Temporal Gyrus | 42 15 -30 | 9 | 7.35 | 5.36 | 0.002 |
| Pyramis | 21 -69 -39 | 7 | 6.30 | 4.87 | 0.016 |
| Superior Temporal Gyrus | 39 -33 0 | 4 | 6.27 | 4.85 | 0.017 |
| **neutral faces first > last block** | | | |  |  |
| Middle Occipital Gyrus | 48 -72 0 | 2924 | 15.43 | 7.70 | < 0.001 |
| Superior Temporal Gyrus | 42 15 -30 | 21 | 8.60 | 5.87 | < 0.001 |
| Thalamus | -24 -30 0 | 26 | 7.76 | 5.54 | 0.001 |
| Pyramis | 21 -69 -39 | 13 | 6.58 | 5.00 | 0.009 |
| Precentral Gyrus | -63 -9 -27 | 4 | 6.54 | 4.99 | 0.010 |
| Extra-Nuclear | 30 -33 3 | 7 | 6.25 | 4.84 | 0.018 |
| **non-social positive first > last block** | | | | |  |
| Middle Occipital Gyrus | 51 -72 6 | 14 | 8.25 | 5.73 | < 0.001 |

Table S4 displays the observed whole brain activation patterns after conducting FmL habituation. All contrasts were thresholded at a p < .05 FWE-corrected level.

**Supplementary Methods**

Table S5a-c present the number of runs per category that each participant viewed, because of data loss, participants viewed each category between 6 – 8 times instead of viewing each category 8 times.

Table S5a: Number of blocks per face category seen by each participant

| **Number of blocks per category for each participant** | | | |
| --- | --- | --- | --- |
| **Participant ID** | fearful faces | neutral faces | happy faces |
| **ID_1** | 8 | 8 | 8 |
| **ID_2** | 8 | 8 | 8 |
| **ID_3** | 8 | 6 | 8 |
| **ID_4** | 8 | 7 | 7 |
| **ID_5** | 8 | 7 | 7 |
| **ID_6** | 8 | 7 | 7 |
| **ID_7** | 8 | 7 | 7 |
| **ID_8** | 8 | 7 | 7 |
| **ID_9** | 8 | 7 | 7 |
| **ID_10** | 8 | 7 | 7 |
| **ID_11** | 8 | 6 | 8 |
| **ID_12** | 8 | 7 | 7 |
| **ID_13** | 8 | 7 | 7 |
| **ID_14** | 8 | 6 | 8 |
| **ID_15** | 8 | 6 | 8 |
| **ID_16** | 8 | 7 | 7 |
| **ID_17** | 8 | 6 | 8 |
| **ID_18** | 8 | 6 | 8 |
| **ID_19** | 8 | 7 | 7 |
| **ID_20** | 8 | 7 | 7 |
| **ID_21** | 8 | 7 | 7 |
| **ID_22** | 8 | 6 | 8 |
| **ID_23** | 8 | 7 | 7 |
| **ID_24** | 8 | 7 | 7 |
| **ID_25** | 8 | 7 | 7 |
| **ID_26** | 8 | 6 | 8 |
| **ID_27** | 8 | 7 | 7 |

Table S5b: Number of blocks per social scene category seen by each participant

| **Number of blocks per category for each participant** | | | |
| --- | --- | --- | --- |
| **Participant ID** | negative social | neutral social | positive social |
| **ID_1** | 8 | 8 | 8 |
| **ID_2** | 8 | 8 | 8 |
| **ID_3** | 7 | 7 | 6 |
| **ID_4** | 7 | 7 | 6 |
| **ID_5** | 7 | 7 | 6 |
| **ID_6** | 7 | 7 | 6 |
| **ID_7** | 7 | 7 | 6 |
| **ID_8** | 7 | 7 | 6 |
| **ID_9** | 7 | 7 | 6 |
| **ID_10** | 7 | 7 | 6 |
| **ID_11** | 6 | 8 | 6 |
| **ID_12** | 7 | 7 | 6 |
| **ID_13** | 7 | 7 | 6 |
| **ID_14** | 7 | 7 | 6 |
| **ID_15** | 7 | 7 | 6 |
| **ID_16** | 7 | 7 | 6 |
| **ID_17** | 7 | 7 | 6 |
| **ID_18** | 7 | 7 | 6 |
| **ID_19** | 7 | 7 | 6 |
| **ID_20** | 7 | 7 | 6 |
| **ID_21** | 7 | 7 | 6 |
| **ID_22** | 7 | 7 | 6 |
| **ID_23** | 7 | 7 | 6 |
| **ID_24** | 7 | 7 | 6 |
| **ID_25** | 7 | 7 | 6 |
| **ID_26** | 8 | 7 | 6 |
| **ID_27** | 7 | 7 | 6 |

Table S5c: Number of blocks per non-social scene category seen by each participant

| **Number of blocks per category for each participant** | | | |
| --- | --- | --- | --- |
| **Participant ID** | negative non-social | neutral non-social | positive non-social |
| **ID_1** | 8 | 8 | 8 |
| **ID_2** | 8 | 8 | 8 |
| **ID_3** | 7 | 7 | 8 |
| **ID_4** | 7 | 7 | 8 |
| **ID_5** | 7 | 7 | 8 |
| **ID_6** | 7 | 7 | 8 |
| **ID_7** | 7 | 7 | 8 |
| **ID_8** | 7 | 7 | 8 |
| **ID_9** | 7 | 7 | 8 |
| **ID_10** | 7 | 7 | 8 |
| **ID_11** | 8 | 6 | 8 |
| **ID_12** | 7 | 7 | 8 |
| **ID_13** | 7 | 7 | 8 |
| **ID_14** | 7 | 7 | 8 |
| **ID_15** | 7 | 7 | 8 |
| **ID_16** | 7 | 7 | 8 |
| **ID_17** | 7 | 7 | 8 |
| **ID_18** | 7 | 7 | 8 |
| **ID_19** | 7 | 7 | 8 |
| **ID_20** | 7 | 7 | 8 |
| **ID_21** | 7 | 7 | 8 |
| **ID_22** | 7 | 7 | 8 |
| **ID_23** | 7 | 7 | 8 |
| **ID_24** | 7 | 7 | 8 |
| **ID_25** | 7 | 7 | 8 |
| **ID_26** | 6 | 7 | 8 |
| **ID_27** | 7 | 7 | 8 |

Table S6a presents the interval between the functional and structural scans for each participant in years and table S6b presents the group statistics for this interval in years.

Table 6a: Interval between structural and functional scans for each participant

| **Participant ID** | **Interval in years** |
| --- | --- |
| **ID_1** | 3.47 |
| **ID_2** | 4.18 |
| **ID_3** | 2.67 |
| **ID_4** | 0.86 |
| **ID_5** | 4.59 |
| **ID_6** | 4.36 |
| **ID_7** | 2.04 |
| **ID_8** | 4.62 |
| **ID_9** | 4.35 |
| **ID_10** | 2.45 |
| **ID_11** | 3.63 |
| **ID_12** | 1.58 |
| **ID_13** | 3.61 |
| **ID_14** | 2.23 |
| **ID_15** | 0.07 |
| **ID_16** | 1.06 |
| **ID_17** | 2.44 |
| **ID_18** | 2.66 |
| **ID_19** | 0.53 |
| **ID_20** | 2.03 |
| **ID_21** | 1.61 |
| **ID_22** | 1.84 |
| **ID_23** | 0.23 |
| **ID_24** | 0.50 |
| **ID_25** | 0.23 |
| **ID_26** | 0.21 |
| **ID_27** | 0.10 |

Table 6b: Group statistics for the interval between the structural and the functional scan in years

| **Mean** | **SD** | **Min** | **Max** |
| --- | --- | --- | --- |
| 2.15 | 1.54 | 0.07 | 4.62 |

Abbreviations: standard deviation (SD), minimum (Min), maximum (Max)

***Habituation analysis specifications***

For the habituation analysis, we modeled every block separately. In the Pmod, we used two different approaches: first linear regression and second LOG1 regression. In the LOG1 condition, each block number was added with 1 and then the natural logarithm of this number was taken in order to avoid a logarithm of 1 which equals zero. The FmL analysis includes the first block of the first session and the third block of the second session.

Figure Legends

Figure S1:

Figure S1 presents the stimulus valence ratings for the pictures that are included in this study. The mean category ratings on the left side of each panel stem from the population of the database from which the pictures were taken. The mean category ratings on the right of each panel stem from a group of participants that came to our laboratory.

Figure S2:

Figure S2 presents the stimulus arousal ratings for the pictures that are included in this study. The mean category ratings on the left side of each panel stem from the population of the database from which the pictures were taken. The mean category ratings on the right of each panel stem from a group of participants that came to our laboratory.

Figure S3:

Figure S3 presents the time course extractions of the right amygdala activation for each block. In contrast to the time course extractions of fearful faces, no clear increase for the first or any other block can be observed in the presented conditions.

**Figure S1: valence ratings**

***
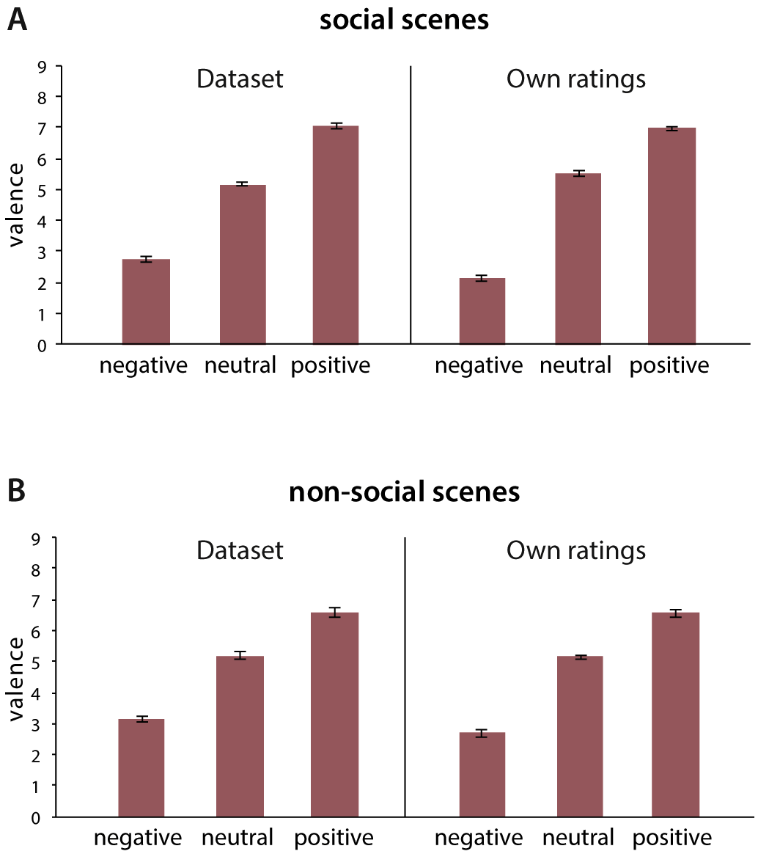
***

**Figure S2: arousal ratings**


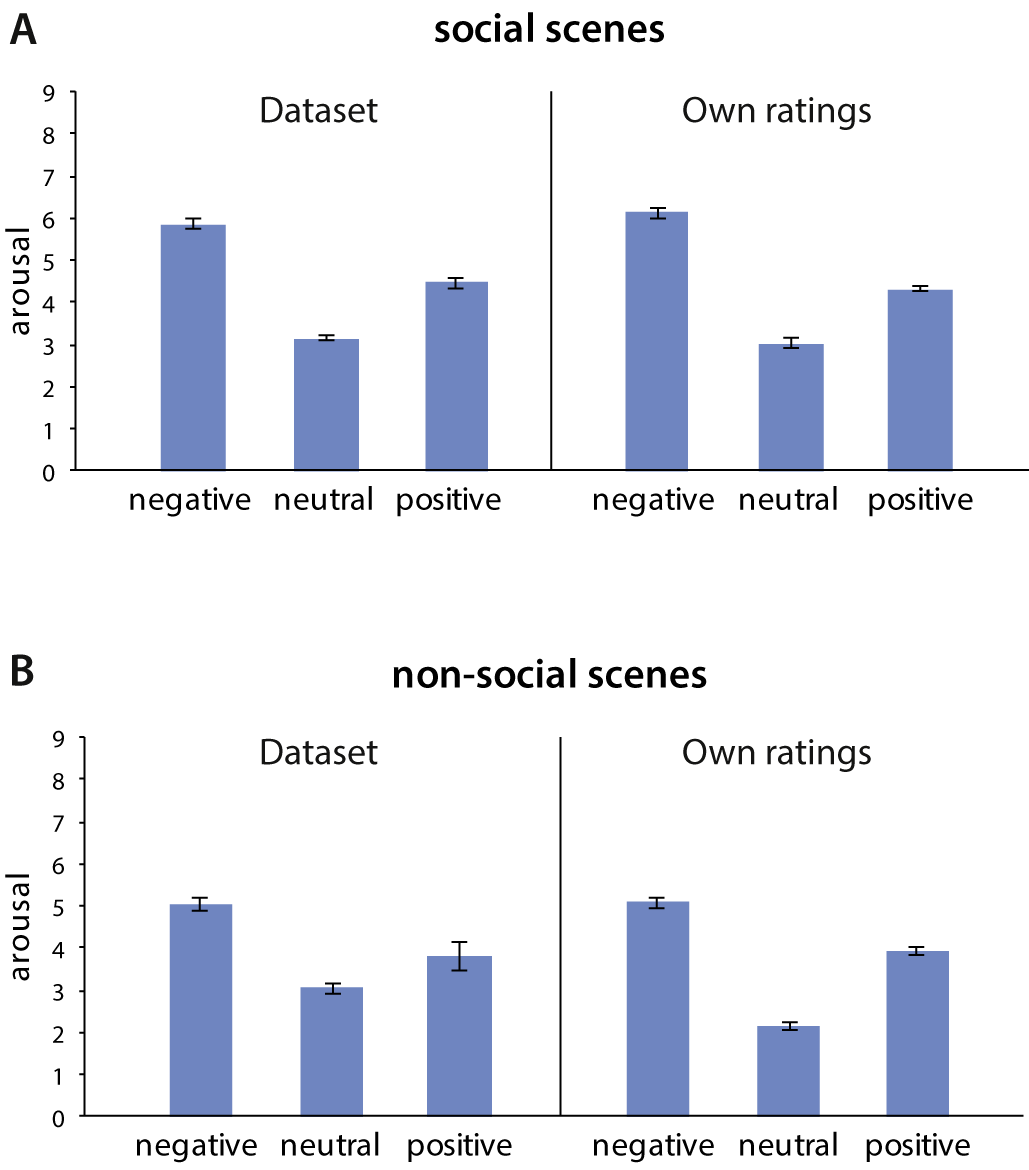


**Figure S3. Time course of amygdala activation for positive faces, negative, neutral and positive social and non-social scenes**


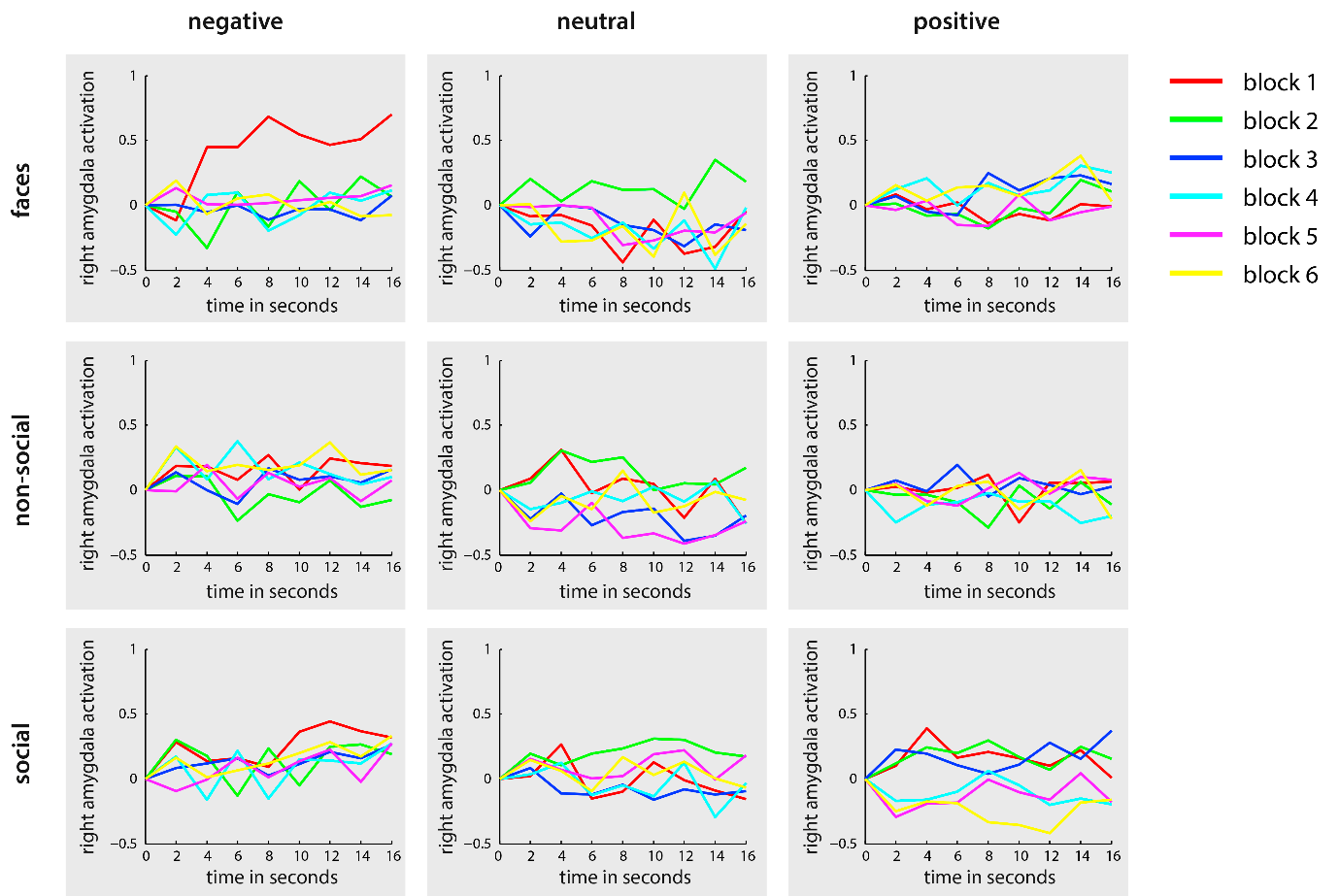

Supplement: Supplementary file 1 — Supplementary Information [file 41598_2018_37163_MOESM1_ESM.docx]
